# Synergistic Inhibition of *Acinetobacter baumannii* Biofilm Formation and Reduction of Lung Inflammation In Vivo by Combination of α-Pinene and Meropenem

**DOI:** 10.3390/microorganisms14050968

**Published:** 2026-04-25

**Authors:** Shengqiang Yang, Yongqi Mu, Lin Wang, Hong Zeng

**Affiliations:** 1 Guangxi Technology Innovation Cooperation Base of Prevention and Control Pathogenic Microbes with Drug Resistance, Youjiang Medical University for Nationalities, Baise 533000, China; yangsq324402@163.com (S.Y.); 18837339823@163.com (L.W.); 2 Key Laboratory of Protection and Utilization of Biological Resources in Tarim Basin of Xinjiang Production & Construction Corps, College of Life Science and Technology, Tarim University, Alar 843300, China; muyq1205@163.com; 3 Bio-Based Active Substances Database Construction and Application Technology Innovation Center, Baise 533000, China

**Keywords:** *Acinetobacter baumannii*, biofilm, α-pinene, meropenem, pneumonia

## Abstract

*Acinetobacter baumannii*, a prominent opportunistic pathogen in healthcare settings, causes severe infections and poses significant challenges for clinical treatment. This study investigates the synergistic effects of α-pinene combined with meropenem (MEM) on *A. baumannii* biofilm formation and lung injury in mice, aiming to develop new strategies to combat persistent infections and antibiotic resistance. α-pinene combined with MEM exhibited strong synergistic antibacterial activity against carbapenem-resistant *A. baumannii* (CRAB 5E9). The combination significantly inhibited biofilm formation, extracellular polymer production, surface motility, and quorum sensing. The expression of key genes such as *ompA*, *bfmR*, *bap*, *csuAB*, *abaI*, and *abaR* was reduced by up to 61%. In vivo, the treatment alleviated weight loss, decreased the bacterial load in lung tissue, and reduced lung inflammation. Furthermore, it significantly suppressed proteins involved in the inflammatory response and the MAPK pathway, including TLR4, NF-κB, NLRP3, TRAF6, ERK2, p38 MAPK, JNK, and TNF-α. The combination of α-pinene and MEM synergistically inhibits *A. baumannii* biofilm formation and alleviates the inflammatory response in a mouse model, offering a potential therapeutic approach for combating *A. baumannii* infections.

## 1. Introduction

*Acinetobacter baumannii,* a Gram-negative bacterium, is a prominent opportunistic pathogen in hospital settings and is a member of the “ESKAPE” group of multidrug-resistant pathogens [1]. It is responsible for a variety of severe healthcare-associated infections, including pneumonia, bacteremia, meningitis, and urinary tract infections, particularly in intensive care units [2,3]. *A. baumannii* exhibits remarkable resistance to multiple antibiotic classes, including β-lactams, aminoglycosides, and quinolones, through mechanisms such as β-lactamase production, mutations in target genes, efflux pump overexpression, and reduced outer membrane permeability [4]. Multidrug-resistant (MDR) and extensively drug-resistant (XDR) strains of *A. baumannii* are now common, with carbapenem-resistant (CRAB) strains showing resistance rates exceeding 90% in many regions [5,6,7]. The U.S. CDC’s 2019 Antibiotic Resistance Threats report identified CRAB as an urgent public health threat, and the World Health Organization has ranked it among the highest priority pathogens for urgent research and development [8].

Biofilm formation is a major factor contributing to *A. baumannii*’s persistence in clinical environments, with over 65% of nosocomial infections and 80% of chronic infections being attributed to biofilms [9]. *A. baumannii*’s ability to form robust biofilms on medical devices and host tissues is a key factor in its pathogenicity and resistance to treatments [10,11,12]. Biofilm-associated genes such as *csuE*, *pgaB*, *epsA*, *bfmS*, and *ompA* are commonly found in clinical isolates, with nearly 98% of strains carrying multiple biofilm-associated genes. The outer membrane protein OmpA, for instance, plays a critical role in cell adhesion, biofilm formation, drug resistance, and host immune modulation [13,14]. The biofilm-associated protein (Bap) and the BfmR-BfmS signaling system further contribute to biofilm formation, virulence, and antibiotic resistance in *A. baumannii* [4,15,16]. Quorum sensing (QS), a cell-to-cell communication system, also regulates biofilm formation and virulence factor secretion in *A. baumannii* [17]. These factors highlight the urgent need for novel strategies targeting biofilm inhibition, eradication of preformed biofilms, and suppression of quorum sensing to effectively manage *A. baumannii* infections.

Recent studies have shown that combination therapy, which involves using two or more agents, can optimize antibiotic effectiveness and combat resistance [18,19]. α-pinene, a naturally occurring monoterpene found in coniferous trees such as Pinus, Abies, and Picea, has demonstrated promising pharmacological activities, including antimicrobial and antibiofilm effects [20,21,22,23,24]. In a previous study, we found that *A. baumannii* isolates from clinical samples from the Affiliated Hospital of Youjiang Medical University for Nationalities exhibited high resistance to carbapenem antibiotics, particularly MEM (71.2%) [25].

Although several studies have reported the synergistic antibacterial activity of different antibiotics against planktonic *A. baumannii*, the specific effects of α-pinene in combination with meropenem on biofilm formation, particularly by clinically carbapenem-resistant strains, remain largely unexplored. Moreover, whether such a combination can attenuate lung inflammation in vivo has not been previously investigated. Therefore, the present study aimed to fill this gap by evaluating the antibiofilm and anti-inflammatory effects of α-pinene combined with meropenem in both in vitro and in vivo models of *A. baumannii* CRAB 5E9 infection.

## 2. Materials and Methods

### 2.1. Reagents

The RNAprep Pure Cell/Bacteria Kit was purchased from Tiangen Biochemical (Beijing, China). For reverse transcription of total RNA, the BeyoRT™II cDNA First Strand Synthesis Kit (RNase H-) was used, which was obtained from Beyotime (Shanghai, China). Real-time quantitative PCR (qPCR) was performed using the 2 × Q3 SYBR qPCR Master Mix Kit from ToloBio (Nanjing, China). The primary antibodies used included anti-Toll-like receptor 4 Rabbit PAb (TLR4, A5258), p38 MAPK Rabbit pAb (A0227), ERK2 Rabbit pAb (A0229), Phospho-JNK Rabbit pAb (AP0276), TRAF6 Rabbit pAb (A16991), β-actin Rabbit pAb (AC038), Vinculin Rabbit pAb (A14193), and HRP Goat Anti-Mouse IgG1 (AS066), all purchased from ABclonal (Nanjing, China) Technology Co., Ltd. The BCA Protein Assay Kit and FITC-ConA were obtained from Beyotime (Shanghai, China). The Omni-Easy™ One-Step PAGE Gel Fast Preparation Kit (10%), RIPA lysate, Omni-Easy™ Instant Protein Loading Buffer, two-color protein prestaining marker (10–180 kDa), Protein Free Rapid Block Buffer (5×), and ECL™ Femto Light Chemiluminescence Kit were sourced from Epizyme (Shanghai, China). The ELISA kits for Tumor Necrosis Factor-α (TNF-α, MM-0132M1) and Interleukin-6 (IL-6, MM-1011M1) were purchased from Meimian (Shanghai, China).

### 2.2. Bacterial Strains

The CRAB 5E9 strain was provided by the clinical department of the Affiliated Hospital of Youjiang Medical University for Nationalities. Previous studies and screenings have demonstrated that CRAB 5E9 forms a robust biofilm and is resistant to carbapenems, including MEM and imipenem. This strain also expresses the *bla*_NDM-1_, *TEM*, *AmpC*, *KPC*, *bla*_OXA-23_ and *bla*_OXA-51_ resistance genes. *Chromobacterium violaceum* (ATCC 12472) was obtained from the American Type Culture Collection. Both CRAB 5E9 and *C. violaceum* ATCC 12472 were stored at −80 °C, revived on Luria–Bertani (LB) agar, and cultured in LB broth for 6–8 and 16 h, respectively, to allow them to reach the logarithmic growth phase.

### 2.3. In Vitro Effect Studies

#### 2.3.1. Antimicrobial Susceptibility Testing

The minimum inhibitory concentration (MIC) of α-pinene and various antibiotics (including imipenem, MEM, ceftazidime, ceftriaxone, levofloxacin, minocycline, gentamicin, amikacin, cefoperazone/sulbactam, piperacillin/tazobactam, and polymyxin B) against CRAB 5E9 was determined using the broth microdilution method, following the Clinical and Laboratory Standards Institute (CLSI M100-S31) guidelines. Briefly, LB broth was added to a 96-well plate, followed by the drug solution after serial dilution, and then the bacterial suspension was inoculated. The well containing only the medium served as the negative control, while the well without drug treatment served as the positive control. After incubation at 37 °C for 24 h, 1 mg/mL of resazurin dye was added, followed by incubation at room temperature for 1 h. Resazurin is reduced to its pink form by viable cells, while resorufin, which exhibits red fluorescence, is produced. The MIC was defined as the lowest drug concentration at which no color change occurred.

#### 2.3.2. Growth Curve

The clinical strain *Acinetobacter baumannii* CRAB 5E9 was identified using Vitek mass spectrometry (BioMérieux, Rhône, France), and its antimicrobial susceptibility was determined via the Vitek2-compact system [25]. This strain survival was assessed at the MIC, 1/2 MIC, 1/4 MIC, 1/8 MIC, and 1/16 MIC of both compounds. The survival of CRAB 5E9 was monitored every hour over 24 h using a spectrophotometer (BioTek®, Winooski, VT, USA). Concentrations that did not inhibit bacterial growth were selected for further experiments.

#### 2.3.3. Checkerboard Assays

To evaluate the combined effects of antibiotics and α-pinene, the method described by Farhan et al. [26] was employed. Briefly, serial dilutions of the antibiotics and α-pinene were prepared in a 96-well plate, both horizontally and vertically. A logarithmic phase *A. baumannii* bacterial suspension (1 × 10^6^ CFU/mL) was added to each well, and the plate was incubated at 37 °C for 24 h before being stained with resazurin. The fractional inhibitory concentration index (FICI) was used as the evaluation criterion.

FICI index = MIC_AB_/MIC_A_ + MIC_AB_/MIC_B_ = FIC_A_ + FIC_B_

MIC_A_ is the MIC of compound A alone, MIC_B_ is the MIC of compound B alone, MIC_AB_ is the MIC of compound A in combination with compound B, and FIC_A_ is the FIC of compound A and FIC_B_ is the FIC of compound B. Synergism was indicated by an FICI ≤ 0.5, additivity was indicated by an FICI > 0.5 and ≤4, and antagonism was indicated by an FICI > 4. The FICI value represents the average of three independent experiments.

Log-phase CRAB 5E9 was adjusted to 1 × 10^6^ CFU/mL and inoculated into LB broth containing α-pinene (32.8 μg/mL) and MEM (2.5 μg/mL). The control group was untreated, incubated in LB broth only, and was included as a negative control. Inhibition was measured by measuring OD_600_ every hour after incubation at 37 °C. CRAB 5E9 treated with α-pinene and MEM was centrifuged at 12,000 rpm for 5 min. A diluted SYTO 9/PI fluorescent staining solution was then added, and the sample was incubated in the dark for 30 min. After centrifugation at 12,000 rpm for 5 min, the supernatant was discarded, and the bacteria were resuspended for observation using confocal laser scanning microscopy (CLSM).

#### 2.3.4. Biofilm Formation Capacity Determination

The biofilm of CRAB 5E9 was treated with either α-pinene and MEM in combination or each compound alone. Briefly, CRAB 5E9 in the logarithmic growth phase was inoculated into LB broth containing α-pinene (32.8 μg/mL) and MEM (2.5 μg/mL) at a concentration of 1 × 10^6^ CFU/mL. The bacterial solutions were added to a 96-well flat-bottom plate for each treatment group. The control group was left untreated, with LB broth as a negative control. After 48 h of incubation at 37 °C, the culture medium was removed. Free bacteria were then washed away with sterile water, and the biofilm was stained with 0.1% crystal violet. The plates were rinsed twice, air-dried, and the stained biofilm was dissolved in 95% ethanol. Absorbance was measured at 570 nm.

The effect of α-pinene and MEM treatment on biofilm thickness was assessed using CLSM. A cover slip was placed in a 6-well plate, followed by the addition of the treated bacterial solution. The plate was incubated at 37 °C for 48 h. Floating bacteria were gently washed away with sterile water, and the biofilm was stained with a FITC-ConA fluorescent solution for 30 min in the dark. The biofilm thickness was then qualitatively analyzed using CLSM.

#### 2.3.5. Extracellular Polysaccharide and Protein Content Determination

The effects of α-pinene and MEM, used alone or in combination, on exopolysaccharide production by CRAB 5E9 were assessed. Logarithmic-phase CRAB 5E9 was inoculated into LB broth containing α-pinene (32.8 μg/mL) and MEM (2.5 μg/mL) at a concentration of 1 × 10^6^ CFU/mL. After 24 h of incubation, the culture was centrifuged, and the supernatant was collected. The extracellular supernatant was then processed by centrifugation, precipitation, and drying. Distilled water was added to the dried material, and the mixture was thoroughly mixed to obtain a polysaccharide solution. An equal volume of 5% phenol was added to the polysaccharide solution, followed by the addition of concentrated sulfuric acid. The mixture was then cooled to room temperature. Absorbance at 490 nm was measured, and the polysaccharide content was determined using a glucose standard curve.

The extracellular supernatant was prepared as described above. Protein concentration was determined using Coomassie blue staining, and optical density was measured at 595 nm after a 10 min incubation at room temperature. The levels of extracellular proteins were quantified based on a protein standard curve.

#### 2.3.6. Motility Assays of *A. baumannii* 5E9

LB medium containing 0.5% agar was poured into plates, and once cooled to 40 °C, α-pinene (32.8 μg/mL) and MEM (2.5 μg/mL) were added and thoroughly mixed. As a blank control group, 1 µL of logarithmic-phase CRAB 5E9 was inoculated onto a flat surface without any treatment. The plates were incubated at 37 °C for 24 h. The diameter of bacterial movement on the plate was measured to evaluate the impact of α-pinene and MEM on surface motility.

#### 2.3.7. Quorum Sensing Assay

*C*. *violaceum* (ATCC 12472) was used as the target strain. The production of purple pigment, regulated by the quorum-sensing mechanism, was triggered when signaling molecules reached a threshold concentration. In this process, acyl-homoserine lactones (AHLs) bind to the CviR receptor, forming a complex that activates the expression of downstream genes responsible for purple pigment production [27]. The quorum-sensing inhibition effects of α-pinene and MEM were evaluated by measuring the inhibition of purple pigment production.

#### 2.3.8. Quantitative Real-Time PCR

Bacterial RNA was extracted, and the total RNA was subsequently reverse-transcribed into complementary DNA (cDNA). Real-time quantitative PCR was performed using the LightCycler 96 system (Roche, Mannheim, Germany). The expression of six genes—*ompA*, *bfmR*, *bap*, *csuAB*, *abaI*, and *abaR*—was analyzed. Primer sequences for these genes were obtained from previous studies [28,29], as detailed in Table S1. The 16S rRNA gene served as the internal control for normalizing the expression levels of the target genes. Relative gene expression was calculated using the 2^−ΔΔCt^ method.

### 2.4. In Vivo Effect Studies

#### 2.4.1. Murine Model of *A. baumannii* 5E9 Infection

Thirty 6–8-week-old 18–20 g specific-pathogen-free BALB/c mice were selected and randomly assigned to five groups (*n* = 10 each group): a normal group, a MEM (250 µg/20 g) group, an α-pinene (3280 µL/20 g) group, an α-pinene + MEM (250 µg/20 g + 3280 µL/20 g) group, and a control group. Mice were provided free access to food and water and housed in an environment with controlled temperature and humidity. Infection of the lungs was carried out using the CRAB 5E9 strain, as previously described [30]. Mice were immunosuppressed with cyclophosphamide 24, 48, and 72 h prior to infection. For infection, mice were anesthetized with a 20% barbiturate solution, and 40 µL of a bacterial (1.5 × 10^8^ CFU/mL) solution was administered via nasal drip. The infected mice exhibited symptoms including shortness of breath, choking, and coughing. After successful generating the model, from the first to the seventh day of the experiment, the remaining groups were administered their corresponding treatment, except for the normal group, which received 0.9% saline. Mice were subjected to weighing procedures prior to the intragastric administration and were euthanized under anesthesia after the seven-day treatment regimen. The study was conducted in accordance with the ethical approval granted by the Medical Ethics Committee of Youjiang Medical College of Nationalities (Ethical Review No. 2023071101). All animal experiments included in this research have been approved by the aforementioned committee and were carried out in strict adherence to the ethical guidelines and legal frameworks established by the State and the institution’s medical ethics policies.

#### 2.4.2. Bacterial Load of Lung Tissue Assay

Mouse lungs (approximately 30 mg) were excised under sterile conditions and placed in 1.5 mL EP tubes containing 900 µL of sterile water and 4–5 stainless steel beads. The tissue was homogenized using a grinder, appropriately diluted and then plated onto LB agar medium. The plates were incubated for 24 h for bacterial colony enumeration.

#### 2.4.3. Determination of Cytokines in Lungs

The levels of IL-6 and TNF-α in mouse lung tissue were measured using enzyme-linked immunosorbent assays (ELISAs) following the manufacturer’s instructions.

#### 2.4.4. Western Blot Analysis

Lung tissue (approximately 20 mg) was minced using surgical scissors and then homogenized with 200 µL of RIPA Lysis Buffer (Epizyme, Nanjing, China), supplemented with a Protease Inhibitor Cocktail (MedChemExpress, Nanjing, China) at a 1:100 (*v*/*v*) dilution. The tissue was lysed on ice for 30 min, followed by centrifugation at 14,000 g for 20 min at 4 °C. The resulting supernatant was collected. Total protein concentration was measured using the Enhanced BCA Protein Assay Kit (Beyotime, Shanghai, China), and the protein sample was subsequently boiled at 95 °C for 10 min.

For SDS-PAGE (10%), 30 μg of the sample was loaded onto polyvinylidene fluoride (PVDF) membranes (MilliporeSigma, Burlington, ON, Canada). After washing, the membranes were blocked with Protein Free Rapid Block, followed by incubation with the primary antibody for 16 h. The excess primary antibody was removed by washing, and the membranes was incubated with the secondary antibody for 1 h. Chemiluminescence was detected using the Omni-ECL™ Femto Light Chemiluminescence Kit (EpiZyme, Nanjing, China), and the images were captured using ChemiScope Capture software (Clinx Science Instruments, Shanghai, China). Band density in the Western blot was quantified using ImageJ software (1.54m), and the relative protein expression of TLR4 was calculated based on the density. The relative expression of TLR4, TRAF6, p38 MAPK, and Phospho-JNK was normalized to the housekeeping genes β-Actin and Vinculin to enable comparison of target protein levels with internal references (*n* = 3).

#### 2.4.5. Histological Sectioning and HE Staining of Lungs

Fresh lung tissue was collected from mice after euthanasia and placed in a 4% formaldehyde solution (pH 7.4) for fixation at 4 °C for 24 h. The tissue was then dehydrated overnight in a 30% sucrose solution (*v*/*v*) at 4 °C, followed by embedding in paraffin. The paraffin-embedded blocks were sectioned and stained with hematoxylin and eosin (HE). The stained sections were examined under a light microscope (Leica, Wetzlar, Germany).

### 2.5. Statistical Analysis

All quantitative tests were performed in triplicate, and statistical analysis was conducted using GraphPad Prism (Version 9.0, GraphPad Software, San Diego, CA, USA). Group differences were analyzed using one-way analysis of variance (ANOVA), with *p*-values < 0.05 considered statistically significant.

## 3. Results

### 3.1. Synergistic Antibacterial Activity of α-Pinene and MEM Against CRAB In Vitro

The antimicrobial activities of α-pinene in combination with eight antibiotics were evaluated against CRAB 5E9 using checkerboard assays (Table 1). Among the tested antibiotics, α-pinene exhibited the strongest synergistic interaction with MEM, with a fractional inhibitory concentration index (FICI) of 0.375. Similar synergistic effects were observed across 11 additional clinical CRAB isolates, with FICI values ranging from 0.28 to 0.59, confirming the robustness of this interaction (Table 2).

**Figure 1:**
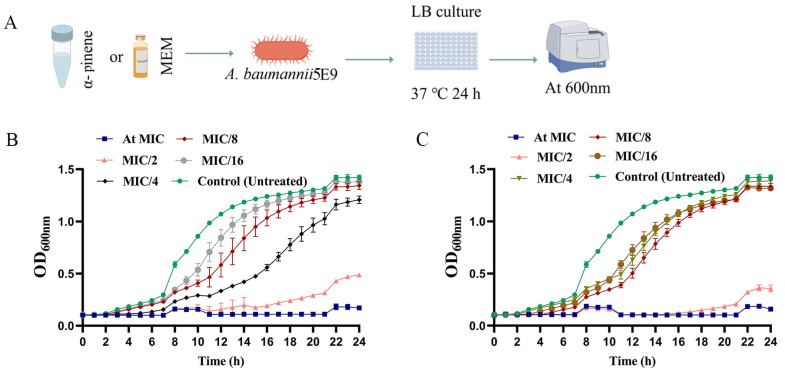
Growth curves of CRAB 5E9 treated with α-pinene and MEM. (**A**) Workflow of MIC determination. (**B**) Growth curve of CRAB 5E9 treated with α-pinene at sub-MICs. (**C**) Growth curve of CRAB 5E9 treated with MEM at sub-MICs.

**Figure 2:**
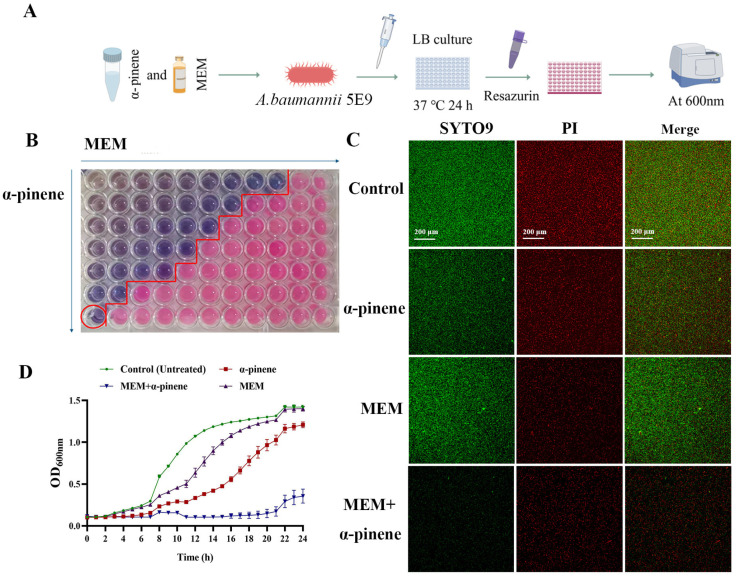
Inhibitory effect of α-pinene combined with MEM on CRAB 5E9. (**A**) Synergistic test of α-pinene combined with MEM on CRAB 5E9. (**B**) The synergistic effect of α-pinene combined with MEM on CRAB 5E9 was detected using the checkerboard assay method. The concentrations of α-pinene and MEM in the red circled holes were 32.80 μg/mL and 2.50 μg/mL, respectively. (**C**) The inhibitory effect of α-pinene combined with MEM on CRAB 5E9 was observed by SYTO9/PI fluorescence staining. (**D**) The time-kill assay was used to observe the inhibitory effect of α-pinene combined with MEM on CRAB 5E9. In the fluorescence staining and time-kill assay test, the concentrations of the α-pinene and MEM doses were 32.80 μg/mL and 2.50 μg/mL, respectively.

The minimum inhibitory concentrations (MICs) of α-pinene and MEM against CRAB 5E9 were 262.8 μg/mL and 10 μg/mL, respectively (Table 1). Then, we examined the effects of α-pinene and MEM on the growth curve of CRAB 5E9 at sub-MICs (Figure 1A). In the control group, the growth curve of CRAB 5E9 displayed typical sigmoidal kinetics, with distinct lag, log, and stationary phases. Both α-pinene and MEM, when applied at 1/2 MIC, delayed the onset of the log phase and inhibited bacterial growth (Figure 1B,C). At sub-inhibitory concentrations (<1/2 MIC), both agents modestly delayed bacterial growth. However, when used in combination, a pronounced inhibitory effect was observed, with bacterial growth reduced by 4–8 fold compared to monotherapy (Figure 2A,B). SYTO9 and PI fluorescent dyes were employed to stain the treated CRAB 5E9, revealing that the combination of α-pinene and MEM significantly inhibited bacterial growth relative to the control group (Figure 2C).

Time-kill assays further demonstrated complete suppression of bacterial proliferation under the combination treatment, indicating a strong synergistic bactericidal effect (Figure 2D). Based on these findings, we selected α-pinene (32.8 μg/mL) and MEM (2.5 μg/mL) for subsequent studies.

### 3.2. Reduction in Biofilm Formation and Down-Regulation of Associated Genes in CRAB 5E9 Due to α-Pinene and MEM Co-Treatment

**Figure 3:**
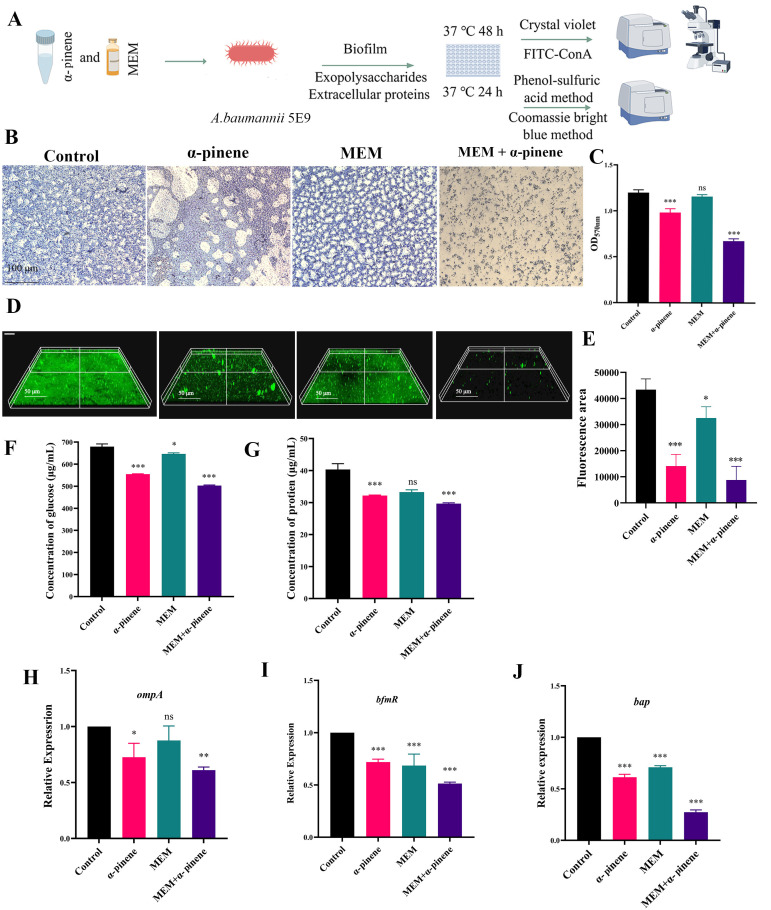
Inhibition of CRAB 5E9 biofilm formation by α-pinene combined with MEM. (**A**) CRAB 5E9 biofilm formation inhibition and membrane permeability assessment. (**B**) Crystal violet staining was used to qualitatively detect effect of α-pinene combined with MEM on CRAB 5E9 biofilm formation. (**C**) Crystal violet staining was used to quantitatively detect effect of α-pinene combined with MEM on CRAB 5E9 biofilm formation. (**D**,**E**) FITC-ConA fluorescence for the qualitative detection of effect of α-pinene combined with MEM on CRAB 5E9 biofilm formation; fluorescent area was analyzed using imageJ. (**F**,**G**) Inhibitory effect of α-pinene combined with MEM on extracellular polysaccharides and extracellular proteins of CRAB 5E9. (**H**–**J**) Inhibitory effect of α-pinene combined with MEM on expression of biofilm-related genes *ompA*, *bfmR*, and *bap* in CRAB 5E9. (**B**–**J**) In the biofilm formation tests, the concentrations of the α-pinene and MEM doses were 32.80 μg/mL and 2.50 μg/mL, respectively. 16S rRNA gene expression was normalized and compared with that of the control group. Error bars represent SD. *: *p* < 0.05; **: *p* < 0.01; ***: *p* < 0.001; ns: *p* > 0.05 compared with the control group.

Biofilms provide a protective niche for *A. baumannii*, enhancing its resistance to external threats [12]. Controlling biofilm formation in *A. baumannii* represents an effective strategy for mitigating its antibiotic resistance. Crystal violet staining of the CRAB 5E9 biofilm treated with α-pinene and MEM revealed significant findings (Figure 3A). Compared to MEM alone, α-pinene was more effective in inhibiting CRAB 5E9 biofilm formation, while MEM treatment alone still allowed for robust biofilm formation. In the α-pinene + MEM group, biofilm formation was reduced by 43.76% and 41.79% compared to the MEM and control groups, respectively (Figure 3B,C). Confocal laser scanning microscopy (CLSM) further confirmed these observations, showing that the biofilm in the control group was thick and compact. In contrast, the biofilm formed after MEM treatment did not significantly differ from the control group, whereas the biofilm treated with α-pinene was noticeably thinner and less dense. The most pronounced effect was observed in the α-pinene + MEM group, where bacterial aggregation was prevented, bacterial populations were substantially reduced, and biofilm formation was significantly diminished (Figure 3D,E).

The extracellular polymers of *A*. *baumannii*, including extracellular polysaccharides and proteins, are key components of the biofilm matrix [31]. Compared to the control group, the α-pinene + MEM combination significantly inhibited the production of extracellular polysaccharides (26.00%) and proteins (26.44%) (Figure 3F,G). Additionally, the α-pinene + MEM treatment led to a marked decrease in the expression levels of the biofilm-associated genes *ompA* and *bfmR*, which are components of a two-component regulatory system (*p* < 0.001). *OmpA* and *bfmR* are essential for the biofilm formation process. The expression of the *ompA* and *bfmR* genes in the α-pinene + MEM group decreased by 38.97% and 48.7%, respectively, compared to the control group (Figure 3H,I). Additionally, the expression of the *bap* gene, which plays a role in biofilm formation and maintenance, was reduced to 27.33% of the level in the control group (Figure 3J).

In conclusion, co-treatment with α-pinene and MEM effectively inhibited biofilm formation and the expression of related genes in CRAB 5E9.

### 3.3. Inhibition of Surface Motility of CRAB 5E9 by Combined α-Pinene and MEM Treatment

**Figure 4:**
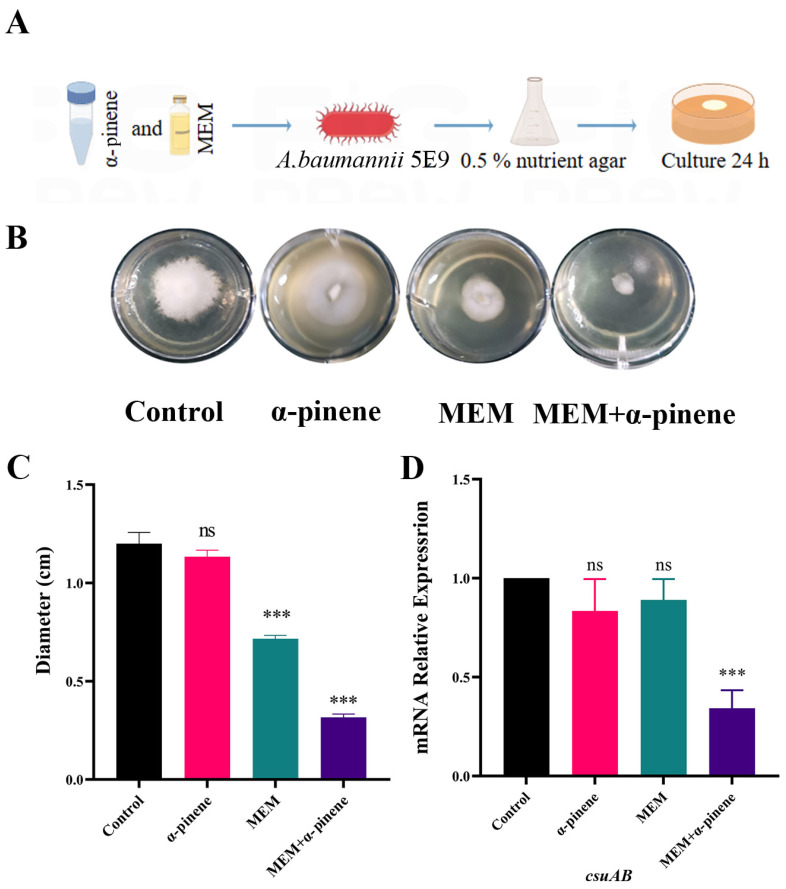
Effect of α-pinene combined with MEM on surface motility of CRAB 5E9. (**A**) Method for studying effect of α-pinene combined with MEM on surface motility of CRAB 5E9. Figure created using Figdraw. (**B**,**C**) Effect of α-pinene combined with MEM on CRAB 5E9 surface motility. (**D**) Inhibitory effect of α-pinene combined with MEM on expression of pilus-related gene *csuAB* in CRAB 5E9. (**B**–**D**) In the surface motility tests, the concentrations of the α-pinene and MEM doses were 32.80 μg/mL and 2.50 μg/mL, respectively. 16sRNA gene expression was normalized and compared with that of the control group. Error bars represent SD. ***: *p*< 0.001; ns: *p* > 0.05 compared with the control group.

Motility in *A. baumannii* plays a crucial role in biofilm formation [32]. The method used to assess surface motility inhibition in CRAB 5E9 is illustrated in Figure 4A. In the control group, the surface motility of CRAB 5E9 was not inhibited, and the diameter of surface movement was approximately 1.20 ± 0.08 cm (Figure 4B). In contrast, the surface motility of CRAB 5E9 in the MEM treatment group was reduced to 0.72 ± 0.02 cm. The α-pinene + MEM combination significantly decreased surface motility by 56.8% compared to the MEM-only group (Figure 4C). This reduction was accompanied by a significant down-regulation of *csuAB*, a gene involved in pilus formation and surface attachment (Figure 4D). These results indicate that co-treatment with α-pinene and MEM effectively inhibited the surface motility of CRAB 5E9.

### 3.4. Inhibition of Quorum Sensing in CRAB 5E9 by α-Pinene and MEM Co-Treatment

**Figure 5:**
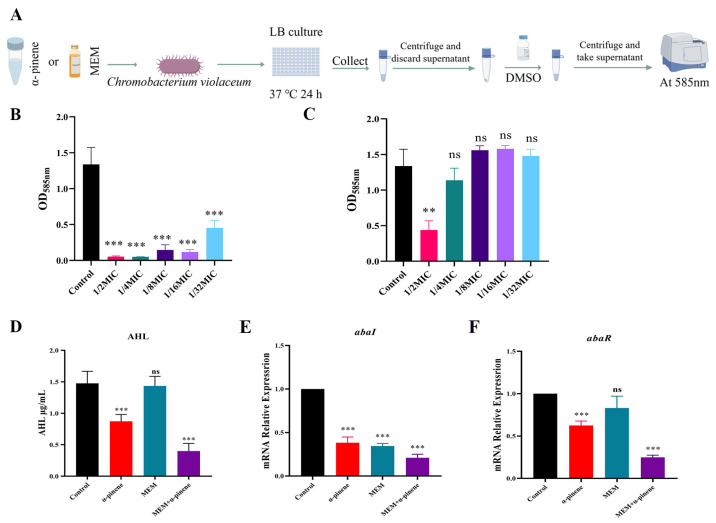
Inhibition of quorum sensing by α-pinene and MEM. (**A**) Method used to measure effect of α-pinene and MEM alone on purple pigment production by *C. violaceum* ATCC12472. Figure created using Figdraw. (**B**) Inhibitory effect of α-pinene on purple pigment production by *C. violaceum* ATCC12472. (**C**) Inhibitory effect of MEM on purple pigment production by *C. violaceum* ATCC12472. (**D**) Effect of α-pinene combined with MEM on AHL production by 5E9. (**E**,**F**) Effect of α-pinene combined with MEM on the expression of 5E9 quorum-sensing-related genes *abaI* and *abaR* in CRAB 5E9. (**B**,**C**) The MIC of α-pinene and MEM against *C*. *violaceum* ATCC12472 were 131.4 μg/mL and 1 μg/mL, respectively. (**D**,**E**). In the tests, the concentrations of the α-pinene and MEM doses were 32.80 μg/mL and 2.50 μg/mL, respectively. Error bars represent SD. **: *p* < 0.01; ***: *p* < 0.001; ns: *p* > 0.05 compared with the control group.

Quorum sensing plays a crucial role in regulating biofilm formation and surface motility in *A. baumannii* [33]. To assess the inhibitory effects of α-pinene and MEM on quorum sensing, we used *C. violaceum* ATCC 12472 as the target strain (Figure 5A). The results showed that α-pinene inhibited purple pigment production more effectively than MEM. At concentrations ranging from 1/2 MIC to 1/16 MIC, α-pinene reduced pigment production by over 90%, and at 1/32 MIC, inhibition remained at 67.4%, which was stronger than the inhibition by MEM at 1/2 MIC (67.2%) (Figure 5B,C). In addition, the acyl-homoserine lactone (AHL) content in the α-pinene + MEM group was significantly reduced compared to the control group (Figure 5D). *AbaI* and *abaR* are key genes involved in quorum sensing in *A. baumannii*. Compared to the α-pinene and MEM groups, the expression of *abaI* and *abaR* genes in the α-pinene + MEM group was significantly reduced to 21.03% and 24.96% of the levels of the control group, respectively (Figure 5E,F).

In vitro experiments showed that the α-pinene + MEM combination reduced biofilm formation, surface motility, and quorum sensing in CRAB 5E9 by down-regulating the expression of biofilm-related genes (*ompA*, *bfmR*, and *bap*), a pilus-related gene (*csuAB*), and quorum-sensing genes (*abaI* and *abaR*).

### 3.5. In Vivo Antimicrobial and Anti-Inflammatory Effects of α-Pinene and MEM on A. baumannii Infections

**Figure 6:**
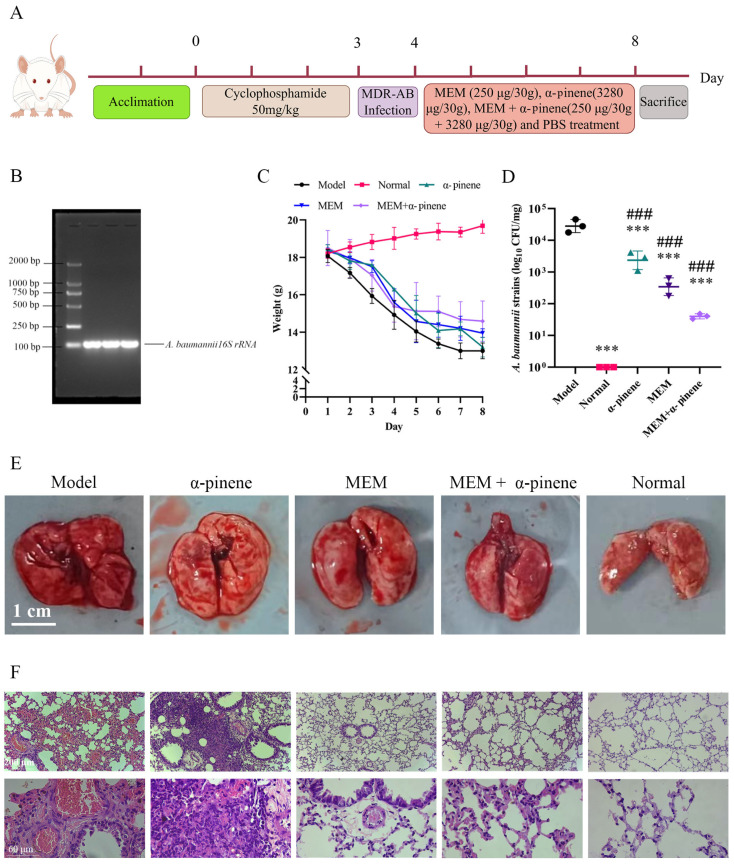
Effect of α-pinene combined with MEM on lung tissue of mice infected with pneumonia caused by CRAB 5E9. (**A**) Flow chart of α-pinene + MEM treatment of pneumonia caused by CRAB 5E9. Figure created using Figdraw. (**B**) Agarose gel electrophoresis pattern for verification of *A. baumannii* infection. (**C**) Weight change of mice infected with pneumonia treated with α-pinene combined with MEM. (**D**) Bacterial load of mice infected with pneumonia treated with α-pinene combined with MEM. (**E**,**F**) Effects of α-pinene combined with MEM on lung tissues after treatment. *** *p* < 0.001 compared with the model group; ### *p* < 0.001 compared with the normal group.

Using a murine pneumonia model is an ideal method for evaluating the effects of drug therapy on *A. baumannii* infections [34]. The protective effects of α-pinene and MEM, both individually and in combination, were assessed in this model (Figure 6A). To confirm the in vivo inhibitory activity of α-pinene combined with MEM against CRAB 5E9, we utilized a murine pneumonia model induced by CRAB 5E9. After euthanizing the mice under anesthesia, lung tissue samples were collected for bacterial analysis. PCR and agarose gel electrophoresis confirmed that the lung tissue contained CRAB 5E9 (Figure 6B). Following infection, the mice exhibited signs of poor health, such as body curling and systemic hypoxia, which slightly improved after drug treatment, though the mice still experienced weight loss (Figure 6C). Mice treated with the α-pinene and MEM combination showed less weight loss. The bacterial load in the lung tissues of the α-pinene + MEM group decreased to 57.01% of that of the model group, while the MEM-only group showed a reduction to 36.04%, indicating a stronger inhibitory effect of the combination treatment on bacterial growth in vivo (Figure 6D).

### 3.6. α-Pinene Combined with MEM Reduces Inflammation Caused by A. baumannii Infection Through MAPK Pathway

Histological examination revealed that the lung tissues of the normal group was smooth and intact, while the model group exhibited hemorrhaging and inflammatory lesions (Figure 6E). Pathological analysis of the model, MEM, and α-pinene treatment groups showed thickened blood vessel walls, vasodilation, congested blood vessels, alveolar rupture, inflammatory cell infiltration, and severe alveolar damage. Notably, neutrophil infiltration in the alveoli of mice treated with α-pinene combined with MEM was significantly reduced, demonstrating the anti-inflammatory and tissue-repair effects of the treatment (Figure 6F).

**Figure 7:**
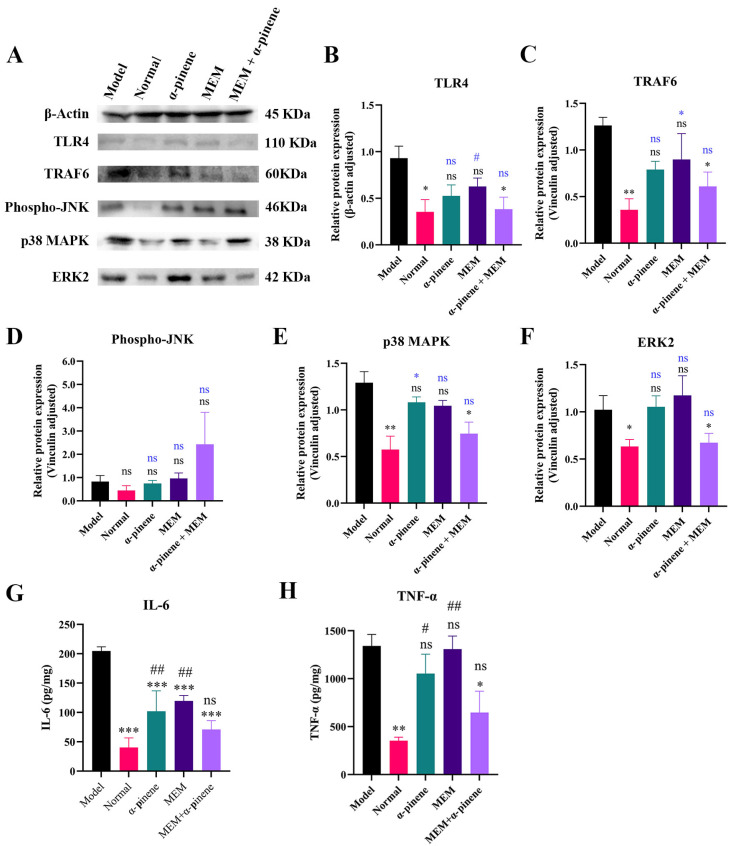
Effect of α-pinene combined with MEM on MAPK pathway in lung tissues of mice. (**A**) Immunoblotting of TLR4, TRAF6, JNK, p38 MAPK, and ERK2 proteins in lung tissue lysates. (**B**–**F**) Western blot analysis of TLR4, TRAF6, JNK, p38 MAPK, and ERK2 in lung tissues. Graphs show quantitative results of gray values after ImageJ analysis. (**G**,**H**) Levels of cytokine IL-6 and TNF-α in lung tissues. Mean ± S.D, *n* = 3. *: *p *< 0.05; **: *p *< 0.01; ***: *p *< 0.001; ns (black): *p* > 0.05 compared with the model group. #: *p *< 0.05; ##: *p *< 0.01; ns (blue): *p* > 0.05 compared with the normal group.

The recognition of bacteria by TLR4 can trigger the overproduction of pro-inflammatory mediators. Specifically, *A. baumannii* releases endotoxins, which bind to the TLR4 receptor and initiate an inflammatory response [35]. MEM combined with α-pinene regulated the expression of key proteins in the MAPK signaling pathway—such as TLR4, TRAF6, p38 MAPK, and ERK2—indicating anti-inflammatory effects (Figure 7A). Compared to the model group, the expression of TLR4 protein was significantly reduced by 58.54% in the α-pinene + MEM treatment group (Figure 7B). The expression levels of TRAF6, p38 MAPK, and ERK2 in the α-pinene + MEM group decreased to 48.27%, 57.67%, and 65.89%, respectively, compared to the model group (Figure 7C,E,F). The JNK signaling pathway plays a pivotal role in inflammation, and increased phosphorylation of JNK indicates that it is activated. α-pinene combined with MEM activated the JNK signaling pathway, inducing the expression of various inflammatory factors and effectively eliminating the toxins produced by *A. baumannii* (Figure 7D). IL-6 and TNF-α are critical pro-inflammatory cytokines involved in both local and systemic inflammatory responses. In this study, enzyme-linked immunosorbent assays (ELISAs) were used to measure the levels of these cytokines in mice. The ELISA results showed that IL-6 and TNF-α levels were 2–3 times higher in the model group compared to the normal group. However, in the α-pinene + MEM treatment group, both cytokines were significantly reduced, with IL-6 and TNF-α levels decreasing to 67% and 48% of the levels of the model group, respectively (Figure 7G,H).

The results demonstrate that the combination of α-pinene and MEM exhibits significant anti-inflammatory effects in vivo, effectively reducing the inflammatory response and tissue damage caused by *A. baumannii* infection.

## 4. Discussion

Carbapenem-resistant *A. baumannii* (CRAB) has become a critically important nosocomial pathogen due to its ability to accumulate multiple resistance determinants and survive under extreme environmental stress. Recent studies suggest that the combination of various compounds with antibiotics can produce synergistic or additive therapeutic effects, effectively reducing the required dosage of each drug, decreasing the risk of resistance, and providing new treatment options for drug-resistant bacteria [36,37]. For instance, the combination of polymyxin (colistin) with doripenem, MEM, or chloramphenicol has shown synergy and prolonged bacterial killing compared to monotherapy [38]. Similar synergistic activities have been described for combinations of colistin with geraniol and linalool; these plant-derived bioactives substantially lowered the MICs of colistin and enhanced its biofilm inhibition effect [39]. Other combinations involving non-traditional agents have also been reported. For example, antimicrobial peptides like TP4-3 combined with MEM demonstrated robust synergy against *A. baumannii*, including enhanced antibiofilm activity and lower resistance induction compared to MEM alone [40].

In this study, it was observed that the combination of α-pinene with MEM exhibited a significant synergistic antibacterial effect on CRAB, with a fractional inhibitory concentration (FIC) index ranging from 0.28 to 0.59 across 12 clinical CRAB strains, significant reductions in MIC values, and synergistic bactericidal activity in vitro. Furthermore, our findings support the notion that biofilm formation and biofilm-associated components are potential targets for treating *A. baumannii* infections [4,41,42]. The combined use of α-pinene and MEM effectively inhibited biofilm formation in CRAB strain 5E9, including the suppression of extracellular polysaccharides and protein production, as well as the expression of the *bap* gene.

These phenotypic effects align closely with literature reports on plant-derived compounds that enhance antibiotic activity not only against planktonic growth, but also against biofilms and virulence. For example, combinations of colistin with plant-derived agents such as geraniol or linalool have shown synergistic reductions in biofilm mass and biofilm-associated virulence traits in MDR *A. baumannii*. Moreover, essential oils have been shown to disrupt quorum sensing and biofilm integrity in *A. baumannii*, similar to our findings with α-pinene.

To explore the synergistic effects of α-pinene and MEM, we conducted in vivo experiments using a mouse model of *A. baumannii* lung infection. After 5 days of treatment, the bacterial load in the lung tissues of mice treated with α-pinene combined with MEM was significantly reduced, which slowed weight loss and alleviated symptoms associated with *A. baumannii* infection (CRAB 5E9). Histological examination through HE staining confirmed that α-pinene combined with MEM significantly improved lung inflammation. Previous studies by Tang and Han have shown that modulation of the MAPK pathway can reduce inflammatory responses [19,43]. *A. baumannii* induces inflammation through the release of lipopolysaccharide (LPS), which acts as a ligand for TLR4, activating immune cells and triggering the MAPK signaling pathway and subsequent inflammation [44]. To further elucidate the mechanisms of inflammation regulation, we investigated whether α-pinene combined with MEM modulates MAPK signaling pathways to exert protective and anti-inflammatory effects. Western blot analysis revealed that the expression of TLR4, TRAF6, p38 MAPK, and ERK2 proteins in mice treated with α-pinene and MEM was significantly reduced, indicating that the combination can mitigate inflammation via the inflammasome and MAPK pathways in vivo. In a similar vein, paeoniflor in combined with luteolin alleviates LPS-induced acute lung injury by modulating MAPK signaling and reducing the expression of p38 and ERK proteins [45].

α-pinene combined with MEM can alleviate the pneumonia inflammation caused by *A. baumannii* infection in vivo.

There are limitations to this research. α-pinene, like many monoterpenes, is susceptible to oxidation and volatilization, and its oral bioavailability is generally low. Formulation strategies such as encapsulation in liposomes, nanoemulsions, or cyclodextrin complexes could improve its stability and bioavailability. Our findings should be interpreted with this consideration, and further pharmacokinetic studies are needed to optimize the delivery of α-pinene for potential therapeutic use.

Studies evaluating the effects of α-pinene and meropenem alone or in combination on healthy (non-infected) animals to fully assess their safety profile are also warranted.

Another limitation of our study is the absence of a clinically relevant positive control antibiotic (for example, colistin or tigecycline) in the experimental design. Future studies comparing the α-pinene + meropenem combination with standard-of-care antibiotics would help better define its potential clinical utility.

## 5. Conclusions

**Figure 8:**
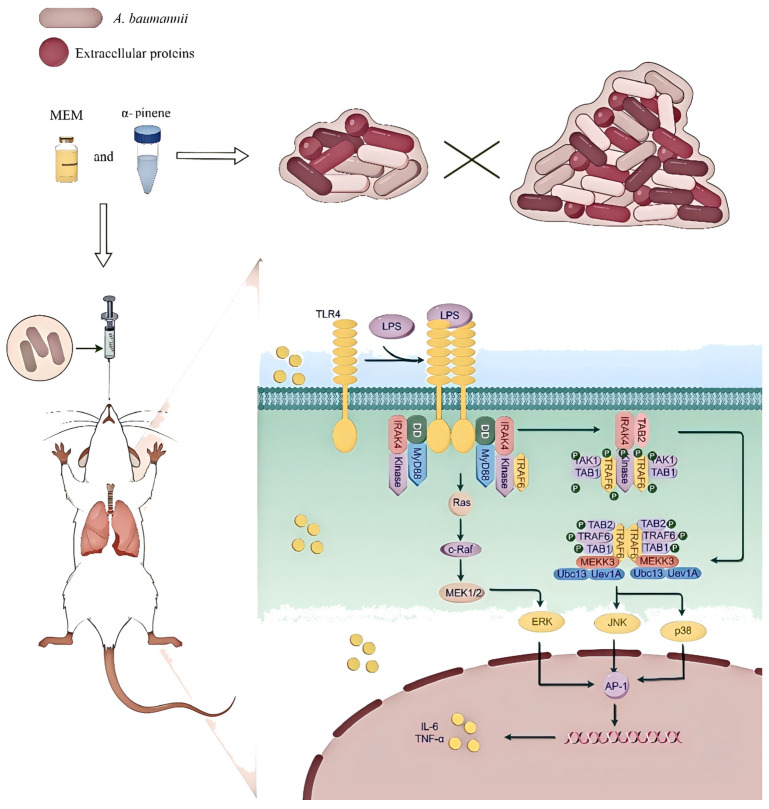
Schematic representation of the therapeutic effect of α-pinene and MEM on pneumonia caused by CRAB 5E9 infection in vivo.

In summary, the combination of α-pinene and meropenem demonstrates a promising synergistic effect in combating CRAB. This combination not only enhances the antimicrobial activity of meropenem, but also significantly reduces key phenotypic traits associated with virulence, including biofilm formation, surface motility, and quorum sensing. Importantly, the dual action of α-pinene—both as an antimicrobial agent and a modulator of host inflammatory responses—was evident in the in vivo mouse model, where it mitigated *A. baumannii*-induced pneumonia through the MAPK signaling pathway. These findings highlight the potential of combining traditional antibiotics with natural compounds, offering a novel and effective therapeutic strategy against MDR and XDR *A. baumannii* infections (Figure 8).

Given the increasing prevalence of multidrug-resistant pathogens, the approach outlined in this study may serve as a valuable alternative to conventional monotherapies, potentially reducing the risk of resistance development and improving patient outcomes. Further investigations, including clinical trials and pharmacokinetic studies, are warranted to fully explore the therapeutic potential of this combination in treating severe *A. baumannii* infections.

## Figures and Tables

**Table 1:** MIC and FIC indices of nine antibiotics and α-pinene, alone and in combination, against CRAB 5E9.

Strain	Compound(s)	MIC Alone (μg/mL)	MIC of Combination of Antibiotic and α-Pinene (μg/mL)	MIC Alone/MIC of Combination	FIC	FICI	Effect
CRAB 5E9	Meropenem	10.00	2.50	4.00	0.25	0.375	Synergistic effect
	α-Pinene	262.80	32.80	8.00	0.125		
	Cefoperazone/ sulbactam	100.00	37.50	2.67	0.38	0.44	
	α-Pinene	262.80	16.40	16.00	0.06		
	Imipenem	200.00	50.00	4.00	0.25	0.50	
	α-Pinene	262.80	65.70	4.00	0.25		
	Piperacillin/ tazobactam	200.00	150.00	1.33	0.75	1.00	Additive effect
	α-Pinene	262.80	65.70	4.00	0.25		
	Minocycline	4.00	3.00	1.33	0.75	1.25	
	α-Pinene	262.80	131.40	2.00	0.50		
	Levofloxacin	25.00	31.25	0.80	1.25	1.26	
	α-Pinene	262.80	4.10	64.00	0.01		
	Ceftazidime	100.00	300.00	0.33	3.00	3.25	
	α-Pinene	262.80	65.70	4.00	0.25		
	Ceftriaxone	100.00	500.00	0.20	5.00	5.50	Antagonistic effect
	α-Pinene	262.80	131.40	2.00	0.50		

**Table 2:** MIC and the FIC index of α-pinene and MEM, alone and combination, against 11 strains of CRAB.

Strain	Compound(s)	MIC Alone (μg/mL)	MIC of Combination (μg/mL)	MIC Alone/MIC of Combination	FIC	FICI	Effect
8C1	Meropenem	8.00	2.50	3.20	0.31	0.44	Synergistic effect
	α-Pinene	525.60	65.70	8.00	0.13		
5C6	Meropenem	16.00	3.75	4.27	0.23	0.30	
	α-Pinene	525.60	32.85	16.00	0.06		
5C7	Meropenem	16.00	3.75	4.27	0.23	0.36	
	α-Pinene	525.60	65.70	8.00	0.13		
8D1	Meropenem	8.00	1.25	6.40	0.16	0.28	
	α-Pinene	525.60	65.70	8.00	0.13		
8F9	Meropenem	4.00	1.25	3.20	0.31	0.44	
	α-Pinene	525.60	65.70	8.00	0.13		
5G5	Meropenem	16.00	2.50	6.40	0.16	0.41	
	α-Pinene	525.60	131.40	4.00	0.25		
5G6	Meropenem	8.00	3.13	2.56	0.39	0.45	
	α-Pinene	525.60	32.85	16.00	0.06		
5G10	Meropenem	8.00	2.50	3.20	0.31	0.44	
	α-Pinene	525.60	65.70	8.00	0.13		
8D2	Meropenem	4.00	1.25	3.20	0.31	0.56	Additive effect
	α-Pinene	262.80	65.70	4.00	0.25		
5E1	Meropenem	8.00	3.75	2.13	0.47	0.59	
	α-Pinene	525.60	65.70	8.00	0.13		
5E4	meropenem	8.00	2.50	3.20	0.31	0.56	
	α-Pinene	525.60	131.40	4.00	0.25		

## Data Availability

The original contributions presented in this study are included in the article. Further inquiries can be directed to the corresponding authors.

## Supplementary Materials

The following supporting information can be downloaded at: https://www.mdpi.com/article/10.3390/microorganisms14050968/s1, Table S1: PCR primer sequence and product length.

## Abbreviations

The following abbreviations are used in this manuscript:

MEM	Meropenem
CRAB	Carbapenem-resistant *A. baumannii*
MDR	Multidrug-resistant
XDR	Extensively drug-resistant
QS	Quorum sensing
ATCC	American Type Culture Collection
LB	Luria–Bertani
MIC	Minimum inhibitory concentration
CFU	Colony-Forming Units
FICI	Fractional inhibitory concentration index
CLSM	Confocal Laser Scanning Microscopy
AHLs	Acyl-homoserine lactones
ELISA	Enzyme-linked immunosorbent assay
