# Supplementary material for: Synergistic Inhibition of Acinetobacter baumannii Biofilm Formation and Reduction of Lung Inflammation In Vivo by Combination of α-Pinene and Meropenem
Source: Microorganisms. 2026 Apr 25;14(5):968. doi: 10.3390/microorganisms14050968 (PMC13210185; doi:10.3390/microorganisms14050968)
Supplement: Supplementary file 1 [file microorganisms-14-00968-s001.zip › microorganisms-4258289-supplementary.pdf]

Synergistic Inhibition of *Acinetobacter baumannii* Biofilm Formation and Reduction of Lung Inflammation in *vivo* by α-Pinene Combination and Meropenem

Contents

Table S1 PCR primer sequence and product length

Genes	Sequence (5′–3′)	Amplicon size (bp)
16s rRNA	Forward：ACGGTCGCAAGACTAAAACTCA<br>Reverse：GTATGTCAAGGCCAGGTAAGGT	108
csuAB	Forward：ATGCGGTAAATACTCAAGCA<br>Reverse：TCACAGAAATATTGCCACCT	204
ompA	Forward：CTCTTGCTGGCTTAAACGTA<br>Reverse：GCAATTTCTGGCTTGTATTG	192
bfmR	Forward：CTGGTAGGTAATGCAGTTCG<br>Reverse：GAGAGACCCAAACCATAACC	200
Bap	Forward：GTACTCCAGCAACGGTTGTA<br>Reverse：GAAGGATCTGCTGTATTCCA	186
abaI	Forward：AATGCCTATTCCCTGCTCAC<br>Reverse：ATTGCTTCTTGCAGAATTGC	132
abaR	Forward：ACCTCTTGTTTGGTCGAG<br>Reverse:：TCCTCGGGTCCCAATAA	127
